# Fixational Eye Movement Correction of Blink-Induced Gaze Position Errors

**DOI:** 10.1371/journal.pone.0110889

**Published:** 2014-10-21

**Authors:** Francisco M. Costela, Jorge Otero-Millan, Michael B. McCamy, Stephen L. Macknik, Xoana G. Troncoso, Ali Najafian Jazi, Sharon M. Crook, Susana Martinez-Conde

**Affiliations:** 1 Barrow Neurological Institute, Phoenix, Arizona, United States of America; 2 Interdisciplinary Graduate program in Neuroscience, Arizona State University, Phoenix, Arizona, United States of America; 3 Department of Neurology, Johns Hopkins University, Baltimore, Maryland, United States of America; 4 Department of Ophthalmology, State University of New York Downstate Medical Center, Brooklyn, New York, United States of America; 5 Unité de Neuroscience, Information et Complexité (CNRS-UNIC), UPR CNRS 3293, Gif-sur-Yvette, France; 6 School of Mathematical and Statistical Sciences and School of Life Sciences, Arizona State University, Phoenix, Arizona, United States of America; University of Leicester, United Kingdom

## Abstract

Our eyes move continuously. Even when we attempt to fix our gaze, we produce “fixational” eye movements including microsaccades, drift and tremor. The potential role of microsaccades versus drifts in the control of eye position has been debated for decades and remains in question today. Here we set out to determine the corrective functions of microsaccades and drifts on gaze-position errors due to blinks in non-human primates (Macaca mulatta) and humans. Our results show that blinks contribute to the instability of gaze during fixation, and that microsaccades, but not drifts, correct fixation errors introduced by blinks. These findings provide new insights about eye position control during fixation, and indicate a more general role of microsaccades in fixation correction than thought previously.

## Introduction

The role of microsaccades in the control and correction of fixation position has been controversial for over 50 years [1]–[4]. Cornsweet originally proposed that microsaccades serve to re-foveate the target after intersaccadic drifts [5], but was subsequently challenged. By the end of the 1970 s, most of the field agreed that microsaccades were not necessary for the control of fixation position, whereas drift (also called slow control) served that purpose (see [4] for a historical review). This conclusion remained uncontested until the early 2000 s, when new analyses indicated that microsaccades introduce fixation errors on a short timescale, and correct fixation errors on a longer timescale [6].

Research into the mechanisms of microsaccade generation has helped to clarify the role of microsaccades in fixation correction. Current findings support a combined role of neural noise and fixation error in triggering microsaccades during attempted fixation, with the contribution of each signal depending on the magnitude of the gaze position error [7]. For example, if a subject’s gaze deviates from the target by ∼0.5° or more, corrective microsaccades might rectify the error [5], [8], whereas if the fixation error is small or insignificant, neural noise might trigger subsequent microsaccades instead [7].

Otero-Millan et al previously showed a corrective role for microsaccades in the form of square-wave jerk pairing [8], [9], but it is not known whether microsaccades play a more general role in error correction during fixation. Indeed, the role of microsaccades in improving fixation stability remains in question [10], as do the comparative roles of drift and microsaccades in oculomotor control [4].

Here we set out to a) characterize the fixation errors due to gaze position changes caused by spontaneous blinks, and b) probe the comparative significance of microsaccades and drift for the correction of blink-induced gaze errors. Our results indicate that blinks contribute to gaze instability during fixation (i.e. eye motion during blinks results in sizable fixation errors at the end of the blinks) and that microsaccades correct blink-induced fixation errors better than drifts.

## Methods

### 1.1 Subjects

#### Non-human primates

Eye position was recorded monocularly at 1000 Hz with a scleral search coil [11]–[13]. Recordings included data from five awake adult rhesus macaques (*Macaca mulatta*). Three monkeys were studied at Harvard Medical School (eye-tracking equipment by Remmel Labs, Inc) and two monkeys were studied at the Barrow Neurological Institute (eye-tracking equipment by Riverbend Instruments, Inc). Standard sterile surgical techniques, recording procedures and animal care methods were approved by the Harvard Medical School Standing Committee on Animals and the Institutional Animal Care and Use Committee at the Barrow Neurological Institute. Monkeys sat in a custom primate chair with their heads restrained, and fixated their gaze on a small fixation target on a video monitor (Reference Calibrator V, 60–120 Hz refresh rate; Barco) placed at a distance of 57 cm. Fruit juice rewards were provided for every ∼1.5–2 seconds of fixation. Eye movements exceeding a 2×2 deg fixation window were recorded but not rewarded. Three monkeys were tested during previously reported studies that addressed different experimental questions [12], [13].

The animals were bred in captivity and housed individually in non-human primate cages (group 4; dimensions 89 cm width, 147 cm height, 125 cm depth, including perch) for the duration of the experiment. Monkeys were provided with several kinds of environmental enrichment, including a television, various fruits and vegetables, food puzzles, perches, Kong toys, mirrors, and other enrichment tools as available, along with visual and auditory contact with several other monkeys that were also housed individually in the same room, and positive daily human contact. The room had a 12 hour light/dark cycle. Regular veterinary care and monitoring, balanced nutrition, and sensory and social environmental enrichment were provided in accordance to the National Institutes of Health Guide for the Care and Use of Laboratory Animals, to maximize physical and psychological well-being. Monkeys had abundant access to food (i.e. feed biscuits were provided twice a day (approximately 12 biscuits/monkey), Purina Lab Diet Monkey Diet, Product# 0001333). Daily fluid intake was controlled and monitored during the experiments. Monkeys typically earned over 80% of their daily fluid allotment during the testing sessions, and received water and/or fruit supplements after the experiments. Whenever the animals were not actively participating in testing or training sessions (i.e. weekends, analysis and manuscript writing periods, etc), they had free access to water in the vivarium.

Cranial head-post and scleral search-coil implantation surgeries were conducted previous to the eye movement recordings, under general anesthesia using aseptic techniques, and with full post-operative analgesia and antibiotic therapy. No animals were sacrificed at the end of the experiments.

#### Humans

We recorded the eye movements of sixteen naive adult subjects (12 males, 5 females) with normal or corrected-to-normal vision, over 4 experimental sessions of ∼30 min each, as part of a previously reported study [14]. Experiments were carried out under the guidelines and ethical approval of the Barrow Neurological Institute’s Institutional Review Board (protocol number 04BN039). Written informed consent was obtained from each subject, and each subject received $15/session. Subjects rested their forehead and chin on the EyeLink 1000 (SR Research) head/chin support 57 cm away from a linearized video monitor (Barco Reference Calibrator V, 75 Hz refresh rate). Subjects were instructed to look at a central circular target, or at the center of a 50% gray screen (see [14] for details). Trials were 30 s long, and subjects took short (∼2–5 min) breaks after each eleventh trial. Each subjects’ eye position was calibrated at the beginning of the experimental session, and re-calibrated after each break. We used custom code and the Psychophysics Toolbox [15]–[17] to display visual stimuli. Six subjects were discarded because they made fewer than 100 blinks. The present analyses do not overlap with analyses previously reported.

### 1.2 Blink detection

#### Non-human primates

**Figure 1:**
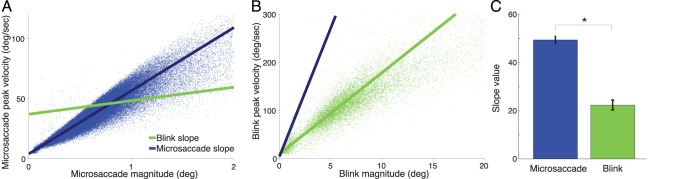
Microsaccadic magnitude/peak velocity relationships. (**A**) and blink magnitude/peak velocity relationship (**B**) for one experimental subject (monkey Y). Each dot represents one microsaccade or blink. The lines are the linear fits to the data. Microsaccadic peak velocity was greater than blink peak velocity. (**C**) Average slope for microsaccades and blinks. Error bars indicate the SEM across subjects. Asterisk indicates statistical significance (two-tailed paired *t*-test, *p<*0.05) (*n* = 5 monkeys).

We identified eye movements during blinks as epochs with sustained motion faster than typical drifts. Specifically, we classified an eye movement sample as part of a blink if 70% of the samples in the 200 msec around it had velocities above a threshold of 3 deg/s plus twice the median velocity of the recording. We added an extra 50 msec before and after each blink to account for the slow start and end of some blinks. To calculate the eye velocity associated with blinks, we low-pass filtered the eye position (50 Hz Butterworth filter of order 20), and then calculated the polar velocity and filtered it with a 21 msec boxcar filter. We defined the magnitude of an eye movement during a blink as the maximum excursion of gaze direction from the initial gaze position at the start of the blink to the final gaze position at the end of the blink (microsaccade and drift magnitudes were calculated similarly). We analyzed eye movements of all magnitudes during blinks. Blinks and microsaccades followed different magnitude/peak velocity relationships (i.e. microsaccades had higher peak velocities than blinks), suggesting that our detection algorithm distinguished blinks from microsaccades successfully (**Figure 1**). The blink magnitude/peak velocity relationship found here is moreover in agreement with that previously reported by [18].

#### Humans

We identified blink periods as the portions of the EyeLink 1000 recorded data where the pupil information was missing. We added 100 ms before and after each period to further include the initial and final parts of the blink, where the pupil is partially occluded. We moreover removed those portions of the data corresponding to very fast decreases and increases in pupil area (>50 units per sample) plus the 100 ms before and after. Such periods are probably due to partial blinks, where the pupil is never fully occluded (thus failing to be identified as a blink by the EyeLink 1000 software) [19].

### 1.3 Saccade detection

We identified saccades with a modified version of the algorithm developed by Engbert & Kliegl [20]–[24] with λ = 8 (used to obtain the velocity threshold) and a minimum saccadic duration of 8 msec, in both humans and non-human primates. Additionally, we imposed a minimum intersaccadic interval of 20 msec so that potential overshoot corrections might not be categorized as new saccades. We defined microsaccades as saccades with magnitude <2° [14], [25]–[27].

### 1.4 Drift detection

We only conducted drift analyses for the search coil-recorded non-human primate data. We defined drifts as the data between (micro)saccades, overshoots, and blinks, occurring in periods of at least 100 msec. To calculate the properties of each drift period, we low-pass filtered the eye position (60 Hz, Butterworth filter of order 13) and removed 10 msec at the beginning and the end to reduce edge effects due to the filter. We defined drift direction and magnitude as the direction and magnitude of the vector between the start and end of each drift.

### 1.5 Fixation error and correction ratio

**Figure 2:**
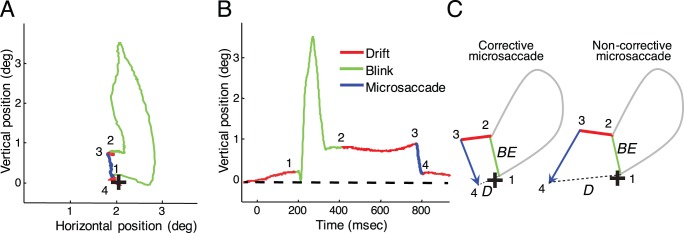
Blink-induced fixation errors. (**A**) Eye position trace showing an example of a blink (green) followed by drift (red) and then a microsaccade (blue). The black cross represents the fixation target. The eye position at the end of the blink (2) does not match the fixation target (black cross). A microsaccade corrects the error by bringing the eye from position (3) to (4), closer to the fixation target. (**B**) Vertical eye position for the same trace. The dashed line represents the fixation target. (**C**) Cartoons of corrective and non-corrective microsaccades. BE (dashed line) indicates the blink-induced fixation error and D (dotted line) the distance between the eye position at the end of the microsaccade and the fixation target. Left: the microsaccade reduces the blink-induced eye position error (D is shorter than BE). Right: the microsaccade increases the eye position error (D is longer than BE).

We defined fixation error as the distance between the current eye position and the position of the fixation target. To quantify how well microsaccades and drift corrected fixation errors due to blinks, we defined the correction ratio (CR) as follows. For each blink and subsequent microsaccade/drift pair (that is, for the first microsaccade and drift that occurred immediately after the end of each blink), we calculated two quantities: BE, the blink error, which is the distance between the fixation point and the eye position at the end of the blink (the distance between point 1 and point 2 in Figure 2B, and the distance between point 2 and the black cross in **Figure 2C**), and D, the distance between the fixation point and the eye position at the end of the first microsaccade/drift following the blink (the distance between point 4 and the black cross for microsaccades, and the distance between point 3 and the black cross for drifts, in **Figure 2C**). We defined the correction ratio as: 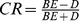. CR is always between −1 and 1, with positive values indicating corrective eye movements (microsaccades/drifts that decrease blink error), and negative values corresponding to non-corrective eye movements (microsaccades/drifts that increase blink error). If the microsaccade/drift brings the eye position to the exact original position then D = 0 and CR = 1.

### 1.6 Permutation analysis

To test the significance of the CR values, we used a permutation analysis to calculate the correction ratio expected by chance. For each blink-induced error we measured CR by replacing the post-blink microsaccade with a randomly selected microsaccade (from all the post-blink microsaccades). That is, we replaced the microsaccade (from point 3 to point 4 in **Figure 2A**) with the new random microsaccade (effectively changing the position of point 4 in **Figure 2A**). Likewise, we replaced each post-blink drift with another random drift. We repeated the permutation method 1000 times and averaged the resulting CR values.

## Results

Awake rhesus macaques fixated a small target while we recorded their eye movements with the scleral search coil technique [11]. During the fixation task, eye movements consisted primarily of microsaccades and drifts, in addition to occasional larger saccades and spontaneous blinks. Spontaneous blinks resulted in fixation errors of moderate size (0.88+/−0.45 deg on average), where the eye position at the end of a blink did not match the position of the fixation target (**Figure 2** **;** **Table 1**). Fixation errors after blinks were generally larger than fixation errors previous to blinks (0.33+/−0.17 deg on average). (See **Table 2** for the average magnitude of fixation errors associated with different types of ocular events).

**Table 1:** Characteristics of non-human primate blinks.

Non-humanprimate	#Blinks	% blinksincreasingfixation error	Blink rate(blinks/sec)	Average magnitudeof blink-inducederror (deg)	Median post-blinkmicrosaccadelatency (msec)
Y	22,686	75.71%	0.28	0.79	408
H	16,404	85.55%	0.23	0.83	345
C	9,797	72.92%	0.28	0.75	215
J	1,928	59.62%	0.19	1.28	202
F	43,700	58.74%	0.39	0.72	154

**Table 2:** Average magnitude of fixation errors induced by different types of ocular events.

	Blinks	Microsaccades	Drifts
**Non-human primates**	0.88+/−0.45 deg	0.44+/−0.15 deg	0.76+/−0.18 deg
**Humans**	0.95+/−0.38 deg	0.68+/−0.16 deg	–

Fixation errors associated with blinks tended to be larger than those associated with (all) microsaccades or drifts, but not significantly so.

We set out to determine whether fixational eye movements (i.e. microsaccades and/or drift) produced after blinks might correct or reduce blink-induced fixation errors. We limited our analyses to those cases where the fixation error increased from pre-blink to post-blink. This occurred for the 70.50% of the non-human primate blinks.

**Figure 3:**
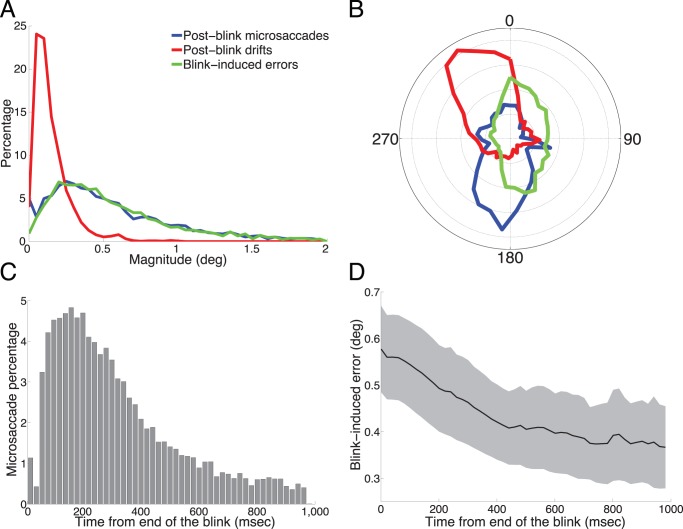
Blink-induced error and microsaccade properties. (**A**) Normalized magnitude distributions of blink-induced fixation errors, post-blink microsaccades, and post-blink drifts across non-human primates. The distribution of post-blink microsaccade magnitudes matches closely that of blink-induced fixation errors. (**B**) Polar histogram of the directions of blink-induced fixation errors, post-blink microsaccades, and post-blink drifts. Blink-induced fixation errors are more likely directed upward. Post-blink microsaccades tend to move the eye downward, thus counteracting the error introduced by the blink. (**C**) Latency distribution for post-blink microsaccades (all monkeys combined): 74.12% of post-blink microsaccade onsets occurred in the initial 400 msec after the end of the blink. (**D**) Blink-induced error as a function of time, from the end of the blink onward. We calculated the blink-induced error at every point in time, whether there were concurrent microsaccades or drifts. The blink-induced error declines gradually, showing the largest decrease in the initial 400 msec interval, simultaneous to the highest production of post-blink microsaccades. Shaded area indicates the SEM across monkeys (*n* = 5 monkeys).

The magnitudes of post-blink microsaccades were significantly correlated to those of blink-induced fixation errors (*p*<10^−13^; *r* ^2^ = 0.075) (**Figure 3A**). Moreover, the direction of post-blink microsaccades was counter to that of blink-induced fixation errors (**Figure 3B**), supporting a potential corrective role for post-blink microsaccades. Microsaccade production was highest (74.12% of all post-blink microsaccades) in the first ∼400 msec after the blink, and asymptoted subsequently (**Figure 3C**). Accordingly, post-blink fixation errors were largest immediately after the blink, and decreased gradually for the first several hundred msec, stabilizing at ∼400 msec after the end of the blink (**Figure 3D**).

We conducted equivalent analyses for human fixational eye movements recorded with a high-speed video-tracker (EyeLink 1000, SR Research; see Methods for further details), and also limited the analyses to those cases where the fixation error increased from pre-blink (0.41+/−0.19 deg on average) to post-blink (0.95+/−0.38 deg on average). This occurred for 61.23% of the blinks in the human data. The results were comparable to those found in non-human primates (**Figure S1**). Microsaccade production was highest (61.32% of all post-blink microsaccades) in the first ∼200 msec after the blink (**Figure S1A**). Correspondingly, blink-induced fixation errors greatly decreased during this period, stabilizing at ∼400 msec after the end of the blink (**Figure S1B**).

**Figure 4:**
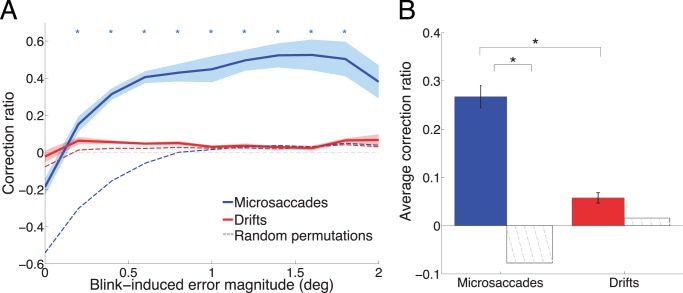
Blink-induced error correction by fixational eye movements. (**A**) Correction ratio for microsaccades and drifts (solid lines) versus random permutations (dotted lines) as a function of error magnitude. Asterisks indicate statistical significance (two-tailed paired *t*-test between microsaccade or drifts and permutations, Bonferroni corrected *p*<0.01). Microsaccades correct blink-induced fixation errors better than chance (i.e. random permutations; see *Methods* for details). Drifts are not significantly different than permutation. Microsaccades corrected large blink-induced errors (>0.2 degrees) better than small blink-induced errors. Error bars and shaded areas indicate the SEM across monkeys (*n* = 5). (**B**) Average correction ratio for microsaccades and drifts (filled bars) compared to chance (striped bars). Asterisks indicate statistical significance (two-tailed paired *t*-test, *p<*0.01) (*n* = 5 monkeys).

To further establish whether fixational eye movements might correct post-blink gaze position errors, we calculated the correction ratio for microsaccades and drifts (CR; i.e. how much microsaccades and drifts reduced (positive CR) or increased (negative CR) such errors; see *Methods*). 76.65% of all post-blink microsaccades and 47.82% of all post-blink drifts reduced blink-induced error (positive CR). To establish significance, we compared those values to a chance level determined by random permutations (see *Methods*), and found that microsaccades, but not drifts, significantly corrected blink-induced errors in primates (**Figure 4**).

In humans, 82.39% of all post-blink microsaccades reduced blink-induced error. Also consistent with the primate results, human microsaccades corrected blink-induced errors significantly better than chance (**Figure S2**). We did not perform drift analyses on the human eye movement data (see *Methods*).

**Figure 5:**
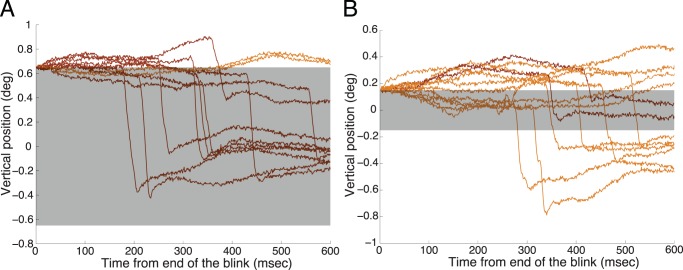
Microsaccades decrease large and increase small blink-induced fixation errors. (**A**) Vertical eye-position traces after 11 randomly-selected blinks that led to large vertical errors ([0.64–0.66 deg], monkey Y). (**B**) Vertical eye-position traces after 11 randomly-selected blinks that led to small vertical errors ([0.14–0.16 deg], monkey Y). (**A, B**) Grey band: range of final eye positions resulting in a positive CR. Brown traces: microsaccades decreased the blink-induced error. Orange traces: microsaccades increased the error. [We note that, although we considered all blinks in our analyses, blinks that took the eye below the fixation point were relatively infrequent (∼18%). Thus, this figure illustrates the more typical situation where blinks induced errors above the fixation point].

Large blink-induced errors (>0.2 deg) were more effectively corrected than smaller ones in the primate (**Figure 4A**). We note that the chance level for the CR is negative (rather than zero) for small blink-induced fixation errors. In the presence of very small errors, microsaccades will tend to be error-increasing rather than error-correcting, owing to being larger than the error. **Figure 5** illustrates blink-induced error magnitude as a major determinant of microsaccade triggering: large or moderate fixation errors resulted in corrective microsaccades (**Figure 5A**), whereas smaller errors led to microsaccades of random directions (**Figure 5B**). In the latter case, microsaccades overshot the zero position typically, even if they traveled in the adequate direction to correct the error (**Figure 5B**).

**Figure 6:**
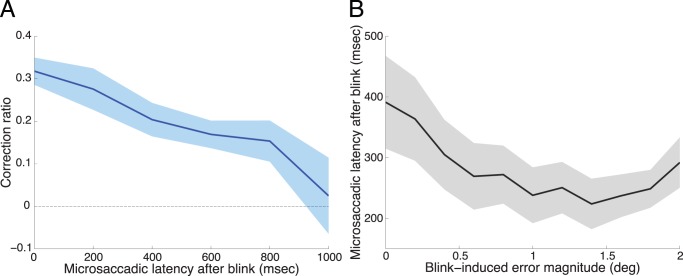
Latency of microsaccades after blinks. (**A**) Relationship between post-blink microsaccadic latency and correction ratio. Microsaccades occurring shortly after blinks were more corrective than microsaccades occurring later in time. (**B**) Relationship between blink-induced error magnitude and post-blink microsaccadic latency. Microsaccade latencies were shorter after large than small errors. Shaded areas indicate the SEM across monkeys (*n* = 5).

If gaze position errors due to blinks trigger corrective microsaccades, then microsaccades occurring shortly after blinks should be more corrective than microsaccades produced later on. Correspondingly, the primate data show that the most corrective post-blink microsaccades had the shortest latencies (**Figure 6A**). Further, microsaccade latencies after blinks were inversely related to the magnitude of the blink-induced errors (**Figure 6B**). That is, microsaccades occurred earlier after large than after small fixation errors due to blinks, and those microsaccades produced shortly after blinks corrected gaze position errors better than microsaccades that occurred later in time.

Equivalent analyses of the human eye movement data showed comparable results (**Figure S3**).

Our combined results indicate that blinks contribute to the instability of gaze during fixation, and that microsaccades help to correct the fixation errors introduced by blinks.

## Discussion

Many microsaccade functions have been proposed, such as keeping the fixated region centered on the optimal locus [5], [6], [14], [28]–[31], counteracting and preventing perceptual fading [19], [26], [32]–[35], enhancing fine spatial detail/improving visual acuity [36], [37], scanning small visual regions [38], [39], and sampling the informative regions of a scene [40]. In addition, microsaccades have been linked to the perception of illusory motion in certain static repetitive patterns [41], [42] (presumably in combination with cortical activation of motion-selective neurons, see [43]–[46]. These various roles need not be mutually exclusive but may overlap considerably).

Here we examined the characteristics of gaze position errors induced by spontaneous blinks in fixating primates and humans, and set out to assess how subsequent fixational eye movements, including microsaccades and drift, might correct them. To our knowledge, no previous studies have determined the comparative contributions of post-blink microsaccades and drifts to the correction of blink-induced errors.

### 3.1 Blink-induced fixation errors

Blinking dynamics have been described in humans and in non-human species [47]–[51]. In the alert cat, spontaneous blinks consist of a fast, large downward lid movement followed by a slower up phase [51]. In humans, transient downward and nasalward movements of both eyes, with amplitudes ranging from one to several degrees, tend to accompany voluntary and reflex blinks [52], [53]. Blink-induced gaze position errors and subsequent corrective saccades have been reported in humans [52], [54] and primates [18]. (Interestingly, strong eye retraction in fish –an early precursor of the eyelid blinks of terrestrial animals– also elicits the return of both eyes to a central position in the orbit [55]). Our present results extend this research by indicating that a) Eye motion during spontaneous blinks results in sizable gaze-position errors in fixating primates and humans, and b) The magnitude of these blink-induced fixation errors (0.88+/−0.45 deg on average in primates and 0.95+/−0.38 deg on average in humans; **Figures 2A–B**, **3D**, **Figure S2,** **Table 1**) is such that microsaccades work to reduce them, and to return the eye to the fixation position.

### 3.2 Corrective role of microsaccades and drift

Having determined that eye movements during blinks result in sizable gaze position errors (**Figures 2A–B**, **3D**, **Table 1**), we set out to measure the comparative contributions of subsequent microsaccades and drift to their correction. Our results show that primate microsaccades work to correct blink-induced gaze position errors during fixation, whereas drifts do not correct better than chance. Our results also indicate that primate and human microsaccades are similarly corrective.

Microsaccades corrected large blink-induced errors better than small blink-induced errors, consistent with the report that large fixation errors (due to error-inducing microsaccades) act to trigger subsequent corrective microsaccades of similar magnitude and opposite direction, resulting in square-wave jerks [8]. Our results are also compatible with the proposal that microsaccades are error-inducing on a short timescale and error-correcting on a longer timescale [6], and indicate that microsaccades play a more general role in error correction during fixation than thought previously.

In sum, our data indicate that 1) blinks contribute to the instability of gaze during fixation in the primate, 2) microsaccades but not drifts correct fixation errors introduced by blinks better than chance, 3) large fixation errors are better corrected than small errors, and 4) that non-human primate and human microsaccades are similarly corrective. These findings provide new insights about eye position control during fixation.

## Supporting Information

Figure S1 **Blink-induced error and microsaccade properties in human subjects.** **(A)** Latency distribution for post-blink microsaccades (all human subjects combined): 61.32% of post-blink microsaccade onsets occurred in the initial 200 msec after the end of the blink. **(B)** Blink-induced error as a function of time, from the end of the blink onward. We calculated the blink-induced error at every point in time, whether there were concurrent microsaccades or drifts. The blink-induced error declines gradually, showing the largest decrease in the initial 400 msec interval, simultaneous to the highest production of post-blink microsaccades. Shaded area indicates the SEM across human subjects (*n* = 11).
(EPS)

Figure S2 **Blink-induced error correction by microsaccades in human subjects.** **(A)** Correction ratio for microsaccades (solid lines) versus random permutations (dotted lines) as a function of error magnitude. Asterisks indicate statistical significance (two-tailed paired *t*-test between microsaccade and permutations, Bonferroni corrected *p*<0.01). Microsaccades correct blink-induced fixation errors better than chance (i.e. random permutations; see *Methods* for details). Microsaccades corrected large blink-induced errors (>0.2 degrees) better than small blink-induced errors (see **Figure 4**). **(A, B)** Error bars and shaded areas indicate the SEM across humans (*n* = 11). **(B)** Average correction ratio for microsaccades (filled bars) compared to chance (striped bars). Asterisks indicate statistical significance (two-tailed paired *t*-test, *p<*0.01) (*n* = 11 subjects).
(EPS)

Figure S3 **Latency of microsaccades after blinks in human subjects.** **(A)** Relationship between post-blink microsaccadic latency and correction ratio. Microsaccades occurring shortly after blinks were more corrective than microsaccades occurring later in time. **(B)** Relationship between blink-induced error magnitude and post-blink microsaccadic latency. Microsaccade latencies were shorter after large than small errors. Shaded areas indicate the SEM across humans (*n* = 11 subjects).
(EPS)
